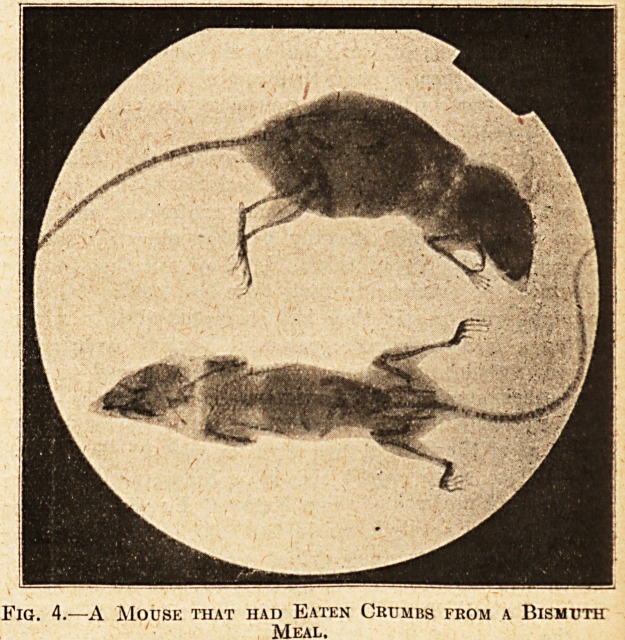# Mr. G. Q. Roberts on St. Thomas's Hospitals History

**Published:** 1918-03-30

**Authors:** G. Q. Roberts


### MR. G. Q. ROBERTS ON ST. THOMAS'S HOSPITAL HISTORY.

### A Few of his Interesting Photographs.

FIG. 1 is a most excellent reproduction of the large photograph of Florence Nightingale which hangs in the dining-room of the Nightingale Home at St. Thomas's Hospital. It is, of course, well known that to Miss Nightingale we are indebted for modern nursing, and St. Thomas's Hospital

FIG. 1.—MISS NIGHTINGALE.
Portrait in Dining Hall, Nightingale Home, St. Thomas's Hospital.

was highly complimented by Miss Nightingale's choice to establish the nurse training-school under the matron, Mrs. Wardroper. When the new hospital was built Miss Nightingale took a very active interest in the plans, and was personally largely responsible for the present form of the Nightingale Home.

Fig. 2. St. Thomas's is one of the great City hospitals, and the Aldermen of the City of London are *ex-officio* governors. They have for hundreds of years filled the offices of president and treasurer of the hospital, but one

FIG. 2.—SIR ROBERT CLAYTON, LORD MAYOR 1680, PRESIDENT OF ST. THOMAS'S HOSPITAL.

of the greatest of all these was Sir Robert Clayton, possibly one of the greatest benefactors of the hospital. When he took office the buildings were in a very bad state of repair, and, in spite of all that had been done to preserve them, were tumbling to pieces. He boldly tackled the matter, and determined in 1693 that the whole hospital should be rebuilt. He himself headed the subscription list with a donation of £600, and by his active exertions, and by his example, succeeded in raising a total sum of £32,000, and carrying out efficiently the rebuilding of the hospital. His name is still perpetuated in Clayton Ward, and his statue stands in the quadrangle of the Medical School.

Fig. 3 is one of the squares which were available for the nurses at a time when there were some intervals of recrea-

FIG. 3.—NURSES ON THE LAWN, NOW OCCUPIED BY HUT FOR SOLDIERS. *Photo*] [*London Photographic Co.*

tion. Now not only have the nurses the heavy demands of war made upon them, but these pleasant quadrangles have for the time being been absorbed and used for the huts, which are proving so extremely useful for the accommodation of soldiers.

Fig. 4. Amongst excellent photographs of *x*-ray work is this, which shows how extremely useful the *x*-rays have proved in diagnostic purposes by the use of a bismuth meal. This poor little mouse had no doubt enjoyed the bismuth crumbs which had fallen on the floor, but in his retreat

FIG. 4.—A MOUSE THAT HAD EATEN CRUMBS FROM A BISMUTH MEAL.

had been caught in a trap, and the happy thought occurred of radiographing him. By this radiogram we see how serviceable the combination of the *x*-ray and the bismuth may prove in diagnosing internal troubles.

## Figures and Tables

**Fig. 1. f1:**
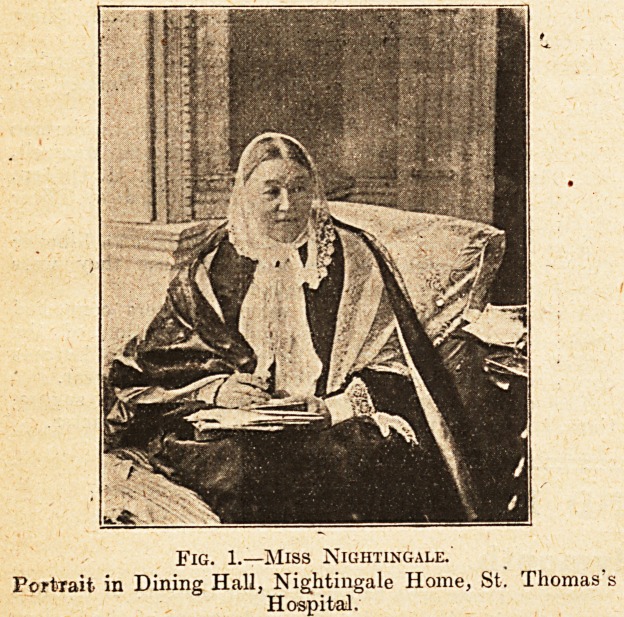


**Fig. 2. f2:**
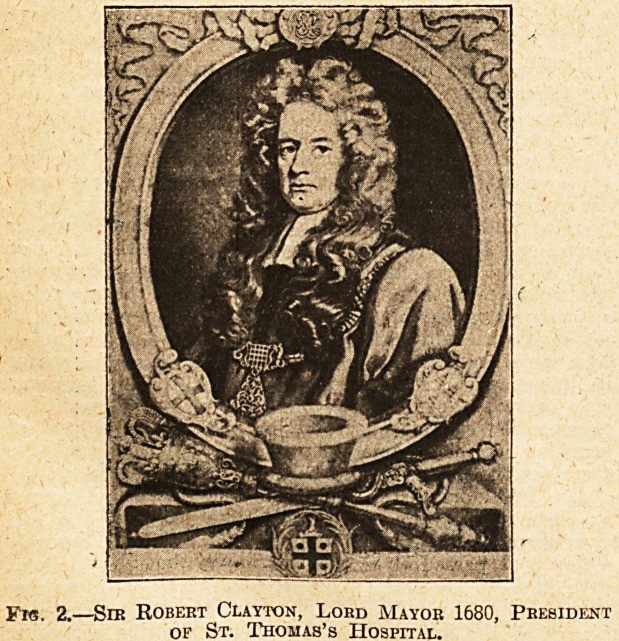


**Fig. 3. f3:**
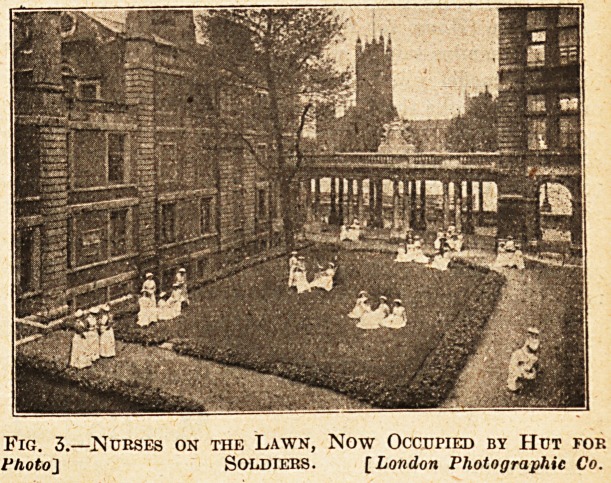


**Fig. 4. f4:**